# Supplementary material for: Staphylococcus Aureus Bacteriuria as a Predictor of In-Hospital Mortality in Patients with Staphylococcus Aureus Bacteremia. Results of a Retrospective Cohort Study
Source: J Clin Med. 2020 Feb 13;9(2):508. doi: 10.3390/jcm9020508 (PMC7074291; doi:10.3390/jcm9020508)
Supplement: Supplementary file 1 [file jcm-09-00508-s001.pdf]

Table S1. Univariate analysis of the whole cohort stratified by in-hospital death.

	Total N = 202	Discharged Alive N = 147	In-Hospital Death N = 55	*p*-Value
	Median (IQR)/% (*n*)	Median (IQR)/% (*n*)	Median (IQR)/% (*n*)	
Age in years	71 (59–80)	70 (59–78)	74 (59–81)	0.300
Male gender	60% (121)	61% (89)	58% (32)	0.872
Onset of SA-BSI after admission in days	2 (1–10)	2 (0–11)	2 (1–10)	0.933
LOS in days	**22 (12–42)**	**28 (15–47)**	**13 (5–28)**	**0.000**
LOS after SA-BSI onset in days	**16 (8–30)**	**18 (11–37)**	**8 (2–19)**	**0.000**
Pitt-Score	**1 (0–4)**	**1 (0–2)**	**2 (1–8)**	**0.000**
Charlson comorbidity index	7 (5–10)	7 (5–9)	7 (6–10)	0.209
SABU	39% (78)	33% (49)	53% (29)	0.012
Myocardial infarction	4% (8)	3% (5)	5% (3)	0.505
Cardiac insufficiency	33% (67)	30% (44)	42% (23)	0.110
Peripheral vascular disease	14% (28)	12% (18)	18% (10)	0.277
Cerebrovascular disease	12% (25)	13% (19)	11% (6)	0.699
Dementia	6% (12)	7% (10)	4% (2)	0.397
Chronic lung disease	25% (51)	29% (42)	16% (9)	0.075
Connective tissue disease	2% (5)	%2 (3)	4% (2)	0.516
Peptic ulcer	4% (9)	6% (9)	–	0.060
Mild liver disease	14% (28)	14% (21)	13% (7)	0.775
Diabetes without complication	28% (56)	32% (47)	16% (9)	0.027
Diabetes with complication	9% (19)	12% (17)	4% (2)	0.086
Renal disease	62% (126)	59% (86)	73% (40)	0.063
Malign tumor	2% (40)	17% (25)	27% (15)	0.103
Moderate to severe liver disease	13% (27)	10% (14)	24% (13)	0.009
Metastatic solid tumor	17% (35)	16% (24)	20% (11)	0.539
AIDS/HIV	1% (1)	–	2% (1)	0.101
Hemiplegia	10% (20)	10% (15)	9% (5)	0.814
Leukemia	–	–	–	not applicable

Lymphoma	0.07 (14)	6% (9)	9% (5)	0.460
SA-BSI with MRSA	25% (50)	25% (37)	24% (13)	0.857
Effective antimicrobial treatment	42% (86)	42% (62)	44% (24)	0.874
Intravenous antimicrobial treatment	97% (195)	97% (143)	95% (52)	0.393
Adaption of Abx without discontinuation	97% (193)	97% (141)	96% (52)	1.000
Spondylodiszitis	2% (4)	1% (2)	4% (2)	0.576
Endocarditis	8% (17)	8% (12)	9% (5)	1.000
Cardiac device	6% (13)	5% (7)	11% (6)	0.193
Artificial heart valve	6% (13)	5% (8)	9% (5)	0.521
Dialysis prior to sampling	15% (30)	12% (18)	22% (12)	0.118
Port catheter prior to sampling	9% (19)	8% (12)	13% (7)	0.416
CVC prior to sampling	31% (63)	32% (47)	29% (16)	0.735
Duration of ICU days	14 (4–27)	15 (3–32)	12 (5–19)	0.341
ICU admission after onset of SA-BSI	61% (124)	55% (81)	78% (43)	0.003
Urinary catheter prior to sampling	49% (98)	44% (65)	60% (33)	0.058
MRSA colonization prior to SA-BSI	27% (54)	28% (41)	24% (13)	0.596
Urine sample from catheter	56% (114)	57% (83)	56% (31)	1.000

Bold parameters represent results with a *p*-value ≤ 0.100. They were further analyzed in the multivariable analyses. Continuous variables are presented as median and interquartile range (IQR). Categorical variables are presented as number (%). Bold values are statistically significant ($p > 0.05$) BSI bloodstream infection, LOS length of stay, CCI Charlson comorbidity index. ABX, antimicrobial therapy.

Table S2. Univariate analysis of the whole cohort stratified by urine positive or negative for Staphylococcus aureus.

	Negative Urine	Positive Urine/SABU	*p*-Value
	Median (IQR)/% (n)	Median (IQR)/% (n)	
Total	100% (124)	100% (78)	not applicable
Age in years	72 (58–80)	70 (59–79)	0.840
Male gender	52% (65)	72% (56)	0.008
Onset of SA-BSI after admission in days	4 (1–11)	1 (0–7)	0.044
LOS in days	27 (15–45)	18 (7–39)	0.004

LOS after SA-BSI onset in days	18 (10–32)	14 (5–28)	0.018
PITT-Score	11 (9–15)	10 (5–14)	0.062
Charlson comorbidity index	7 (5–9)	8 (6–10)	0.008
in-hospital mortality (0 = no;1 = yes)	21% (26)	37% (29)	0.012
Myocardial infarction	5% (6)	3% (2)	0.420
Cardiac insufficiency	38% (47)	26% (20)	0.072
Peripheral vascular disease	15% (19)	12% (9)	0.449
Cerebrovascular disease	12% (15)	13% (10)	0.879
Dementia	7% (8)	5% (4)	0.698
Chronic lung disease	27% (34)	22% (17)	0.370
Connective tissue disease	4% (5)	–	0.073
Peptic ulcer	5% (6)	4% (3)	0.739
Mild liver disease	13% (16)	15% (12)	0.619
Diabetes without complication	31% (38)	23% (18)	0.242
Diabetes with complication	11% (13)	8% (6)	0.508
Renal disease	63% (78)	62% (48)	0.845
Malign tumor	13% (16)	31% (24)	0.002
Moderate to severe liver disease	11% (14)	17% (13)	0.274
Metastatic solid tumor	13% (16)	24% (19)	0.036
AIDS/HIV	0 (0)	1% (1)	0.206
Hemiplegia	7% (8)	15% (12)	0.038
Leukemia	–	–	not applicable
Lymphoma	10% (12)	3% (2)	0.053
SA-BSI with MRSA	24% (30)	26% (20)	0.868
Effective antimicrobial treatment	39% (48)	49% (38)	0.189
Intravenous antimicrobial treatment	96% (119)	97% (76)	0.709
Adaption of Abx without discontinuation	97% (119)	96% (74)	1.000
Spondylodiszitis	2% (3)	1% (1)	0.660
Endocarditis	8% (10)	9% (78)	1.000
Cardiac device	5% (6)	9% (7)	0.378
Artificial heart valve	7% (9)	5% (4)	0,578
Dialysis prior to sampling	15% (18)	15% (12)	1.000
Port catheter prior to sampling	8% (10)	12% (78)	0.462
CVC prior to sampling	35% (43)	26% (20)	0.212
Duration of ICU days	15 (5–26)	13 (2–29)	0.371
ICU admission after onset of SA-BSI	61% (76)	62% (48)	1.000
Urinary catheter prior to sampling	51% (63)	45% (35)	0.470
MRSA colonization prior to SA-BSI	27% (33)	27% (21)	1.000
Urine sample from catheter	60% (74)	51% (40)	0.248

Continuous variables presented as median and interquartile range (IQR). Categorical variables are presented as number (%). BSI bloodstream infection, LOS length of stay, CCI Charlson comorbidity index. ABX, antimicrobial therapy.

Table S3. Multivariable logistic regression for risk factors on SABU.

	p-Value	Odds Ratio	CI95 Low	CI95 High
PITT-Score	0.418	1.046	0.938	1.166
Onset of SA-BSI after admission in days	0.269	0.993	0.981	1.005
Age in years	0.601	0.995	0.976	1.014
Male gender	0.006	2.424	1.297	4.529
Charlson CI	0.019	1.109	1.017	1.210

We calculated odds ratios (ORs) with 95% confidence interval for SABU in our cohort using a stepwise forward approach. For the final model, variables with *p*-values of ≤0.05 were included and variables with $p > 0.05$ were excluded using a stepwise forward approach. All analyses were performed using SPSS (IBM SPSS statistics, Somer, NY, USA) and SAS (SAS Institute, Cary, NC, USA).
